# Point of care airway ultrasound to select tracheal tube and determine insertion depth in cleft repair surgery

**DOI:** 10.1038/s41598-021-84297-4

**Published:** 2021-02-26

**Authors:** Jung Hwan Ahn, Jae Hyun Park, Min Soo Kim, Hyun Cheol Kang, Il Seok Kim

**Affiliations:** 1 Department of Emergency Medicine, Ajou University School of Medicine, Suwon, Republic of Korea; 2 Department of Anesthesiology and Pain Medicine, Kangdong Sacred Heart Hospital, Hallym University Medical Center, Seoul, Republic of Korea; 3 Department of Medical Sciences, Hallym University Graduate School, Chuncheon, Republic of Korea; 4 Department of Applied Statistics, Hoseo University, Asan, Republic of Korea

**Keywords:** Diseases, Risk factors

## Abstract

We aimed to evaluate the efficacy of using airway ultrasonography to select the correct tracheal tube size and insertion depth in pediatric patients who underwent cleft repair surgery as a way to decrease airway complications and adverse events during perioperative periods. Fifty-one patients (age < 28 months) were consecutively divided into conventional (n = 28) and ultrasound (n = 23) groups. Tracheal tube size and insertion depth were determined using the age-based formula and auscultation in the conventional group, whereas using ultrasonographic measurement of subglottic diameter with auscultation and lung ultrasonography in the ultrasound group. We evaluated the initially selected tube size, insertion depth, ventilatory indices, and the incidence of airway complications and adverse events. Tube insertion depth (median [interquartile range]) was significantly greater in the ultrasound group than in the conventional group (13.5 cm [12.5–14.0] vs 13.0 cm [11.8–13.0], *P* = 0.045). The number of complications and adverse events was significantly higher in the conventional group than in the ultrasound group (32.1% vs 4.3%, *P* = 0.013). Airway ultrasound application could reduce airway-related complications and adverse events by determining the appropriate tracheal tube size and insertion depth.

## Introduction

Cleft lip and palate are orofacial deformities that require surgical repair in infancy or early childhood to promote facial growth, speech development, and improve aesthetics^1^. During cleft repair surgery, the risk of airway complications and adverse events is higher due to airway obstruction, difficulty in intubation, and tracheal tube-related problems^2,3^.

Selecting the correct size of the tracheal tube and its placement at the optimal insertion depth are vital aspects of cleft repair surgery^4^. An unsuitably large tube may cause upper airway damage and laryngeal edema followed by post-extubation croup, stridor and a chance of subglottic stenosis. A small and ill-fitting tube may lead to insufficient ventilation and increase aspiration risk and leakage of anesthetic gases into the environment. Repeated laryngoscopies to select the appropriate size of tube may injure the laryngotracheal structures. Accidental extubation and endobronchial intubation caused by displacement of tube may occur intraoperatively, depending on the degree of neck extension and the use of a tongue depressor to improve surgical field exposure^5^.

In children, tracheal tube size and depth are conventionally selected using an age-based formula^6–8^. Studies have suggested positioning the tube by auscultation after endobronchial positioning of the tube tip followed by tube withdrawal to prevent accidental extubation during surgeries performed with neck extension^9^. However, tube selection using the age-based formula is not always appropriate for children with orofacial clefts, because it does not account for individual variability in airway size resulting from delayed growth^10^. Moreover, the age-based formula is calculated using the inner diameter (ID) of tube, while the thickness of tube wall varies for each manufacturer. The outer diameter (OD) of tube is more suitable for sealing the airway and providing effective ventilation. It may be difficult to interpret the underlying pulmonary pathology using auscultation alone, since its sensitivity in discriminating the endobronchial position of tube is only 66%^11^.

Point-of-care ultrasonography has emerged as a powerful modality for perioperative assessment. Tracheal and thoracic ultrasonography can be used for selecting the correct size of tube and verifying its tracheal position^12,13^. The transverse diameter of the subglottic area measured with ultrasonography is more accurate than the age-based formulae for determining tube size in children^14–16^. Pleural sliding and lung expansion on intercostal lung ultrasound or diaphragmatic movement on subcostal diaphragmatic ultrasound may augment determination of tracheal tube position^13^. To the best of our knowledge, there has been no study on choosing an ultrasound-based tracheal tube in cleft repair surgery, which has been reported to have high airway-related complications.

Therefore, this study aimed to compare the efficacy of point-of-care airway ultrasonography with the conventional age-based method to select the correct tracheal tube size and insertion depth as a way to limit airway complications and adverse events during primary cleft repair surgery.

## Materials and methods

### Study design and ethics statement

We retrospectively investigated a prospectively collected data of pediatric patients who underwent primary repair for cleft lip and palate during the period from March 2016 to March 2020 at Kangdong Sacred Heart Hospital, Republic of Korea. All methods were carried out in accordance with relevant guidelines and regulations of the Institutional Review Board of the hospital, which also approved the study (IRB No.: KANGDONG 2020-05-001). The Institutional Review Board of Kangdong Sacred Heart Hospital (Seoul, Republic of Korea) waived the need of a written informed consent. This study was registered with the Clinical Trial Registry of Korea (https://cris.nih.go.kr/cris/index.jsp. KCT0005251).

### Study population

We enrolled fifty-one infants and children younger than 28 months, and the acceptable condition for the surgery was assessed preoperatively by a pediatrician and an anesthesiologist. Subjects who were known to be allergic to the ultrasound gel or had upper or lower respiratory tract infection and an unstable cardiovascular condition were excluded from the study. Consecutively, pediatric patients were divided into the conventional (age-based) group or ultrasound (ultrasound-based) groups for selecting tracheal tube size and verifying its position.

### Methods

Anesthesia was induced with an intravenous bolus dose of 5 mg kg^−1^ thiopental sodium. After loss of consciousness, 0.9 mg kg^−1^ rocuronium was administered to facilitate airway manipulation and tracheal intubation according to the institutional standard. The lungs were ventilated with 100% oxygen through a facemask before intubation. Intubation with direct laryngoscopy with a reinforced cuffed tracheal tube (Mallinckrodt Lo-Contour Oral/Nasal Tracheal Tube Cuffed Reinforced; Covidien, Mansfield, MA, USA) without a Murphy eye was performed in both the groups.

In the conventional group, the size of tube was selected using the Khine formula or Duracher modification as follows: ID (mm) = 0.25 × age (years) + 3.0 or 3.5 and determined by the discretion of the attending anesthesiologist^6,7^. An audible leak at an inspiratory airway pressure of 15–25 cmH_2_O with the tube cuff fully deflated was considered appropriate. The tube was replaced with a half-size (0.5 mm) smaller or larger tube if there was a resistance during tube passage into the glottis, if an audible leak was not detected above 25 cmH_2_O, or if excessive air leakage was observed below 15 cmH_2_O. The insertion depth of tube was estimated using the following formula: depth (cm) = 0.5 × age (years) + 12; and determined by auscultation of bilateral lung sounds. The tip of tube was advanced gently into the right or left mainstem bronchus until the unilateral lung sound was lost. The tube was then withdrawn slowly until bilateral lung sounds could be auscultated and further withdrawn so that the tube tip was positioned 2 cm above the carina^9^. The tube cuff was inflated with a 3 mL syringe to determine the loss of audible leak and cuff pressure was measured using a hand-held manometer (Shiley™ Hi-Lo Hand Pressure Gauge; Covidien, Sulz am Neckar, Germany) limited to 25 cmH_2_O. After positioning the tube with the head in anatomically neutral position, the tube was secured to the midline of the lower lip with an adhesive tape and a sterile strip.

**Figure 1:**
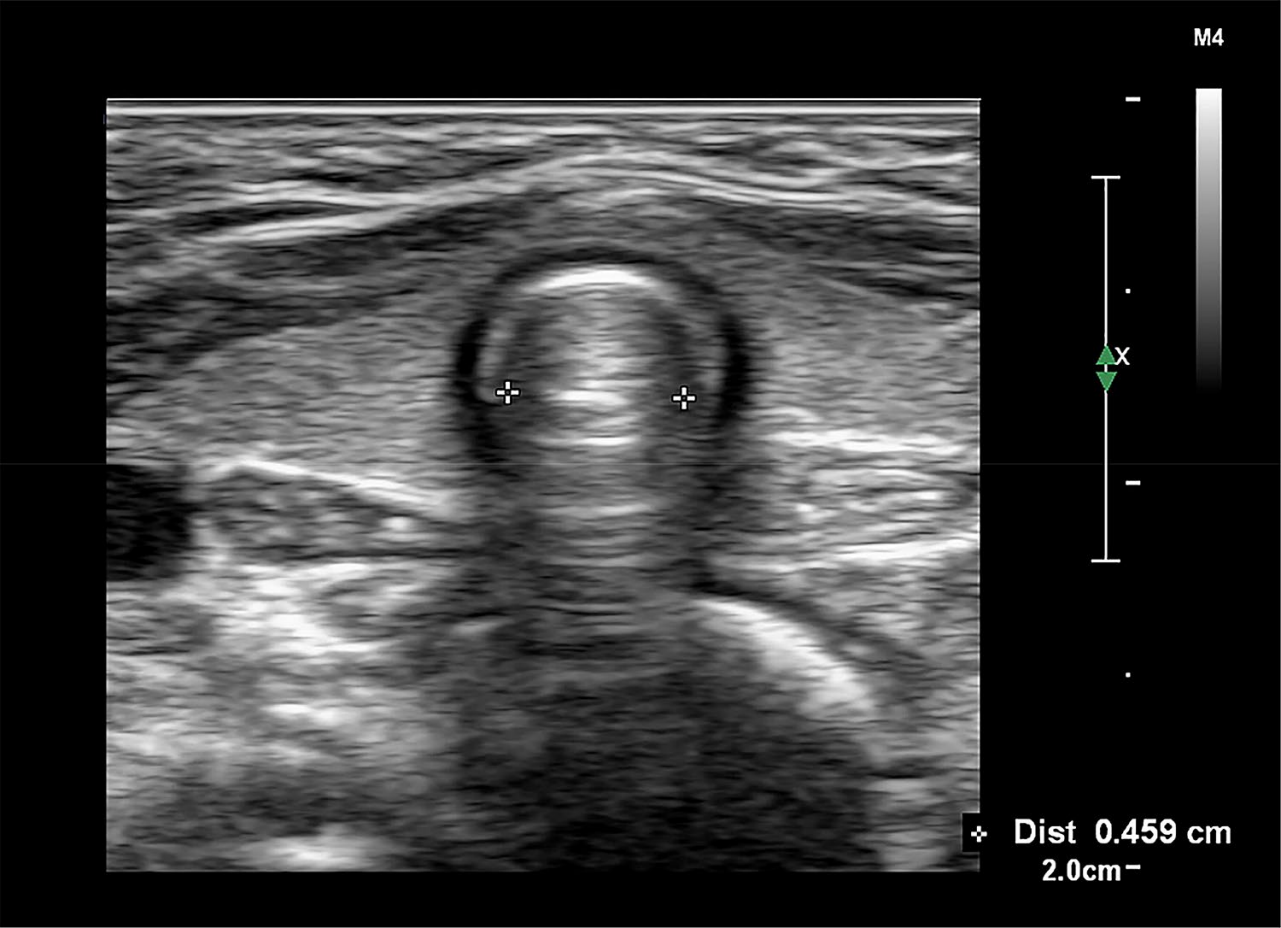
Ultrasonographic measurement of the transverse subglottic diameter of the cricoid cartilage. The cricoid cartilage appears as a round hypoechoic structure with hyperechoic edges, composed of perichondrium. The transverse subglottic diameter is determined by measuring the dimension of the air-mucosa interface (between two crosses) within the bilateral inner margin of the cricoid cartilage.

**Figure 2:**
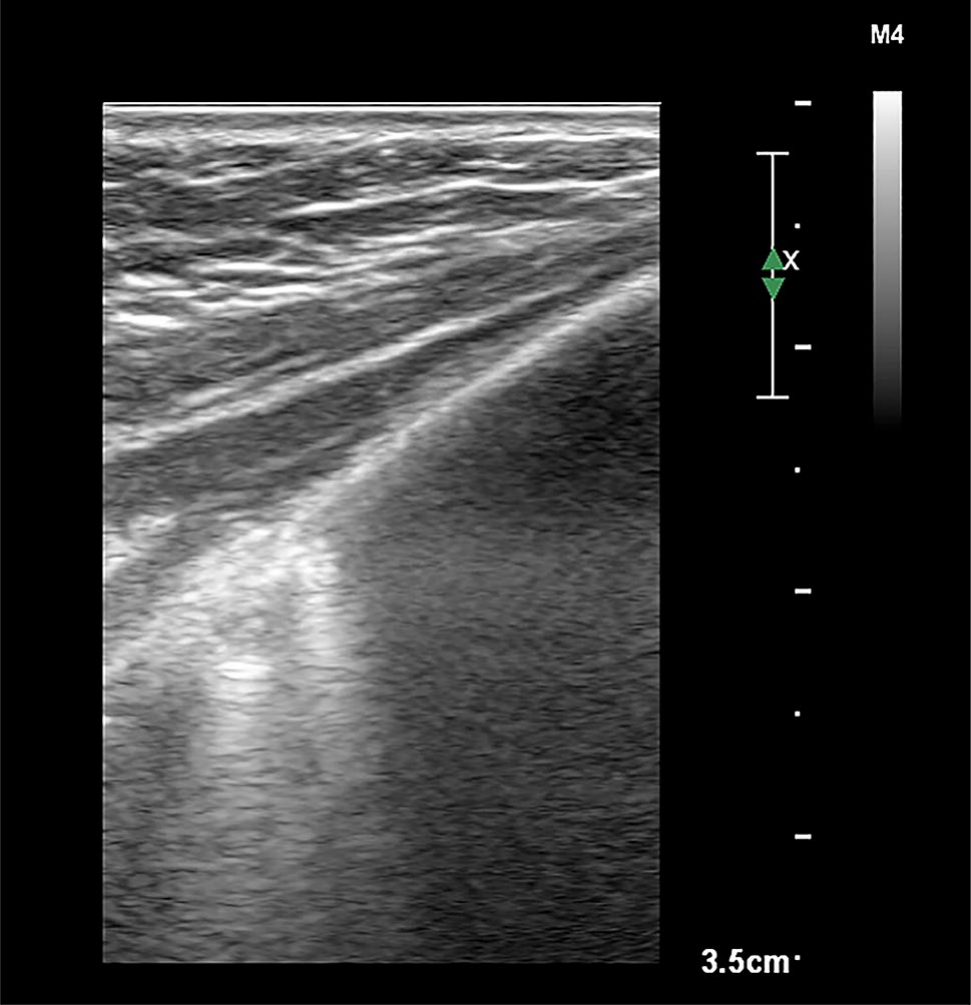
Pleural sliding and lung expansion at the lung-chest wall interface in the intercostal lung ultrasonography image. The pleurae appear as hyperechoic linear structures. Backward and forward horizontal sliding movement of the pleurae is indicated for visceral pleural movement against the parietal pleura, which is synchronized with manual ventilation. Two isolated B-lines arising from the juxta-pleural consolidation are projected vertically and thought to result from anesthesia-induced atelectasis. Statistical analyses were conducted using the SPSS software version 25.0 (IBM Inc., Armonk, NY, USA) (https://www.ibm.com/products/spss-statistics).

In the ultrasound group, a high-frequency hockey stick probe (L15-7io; Phillips, Andover, MA, USA) was placed along the midline of the neck and the transverse diameter of the subglottic area was measured using ultrasonography (Affiniti70; Phillips), with slight extension of the neck before intubation. Facemask ventilation was stopped to limit fluctuation during ultrasound acquisition. The subglottic transverse diameter was measured using the dimension of the air-mucosa interface within the bilateral inner margin of the cricoid cartilage, which appeared as a round hypoechoic structure (Fig. 1). The narrowest subglottic diameter revealed by the three ultrasound images was selected. Subsequently, the make of the tracheal tube, whose OD was smaller than the selected subglottic diameter, was deemed to be the appropriate size. The difference between the two values was less than 0.5 mm to allow free passage of the tube and adequate sealing. Intubation was performed with this size. The insertion depth was determined by auscultation and confirmed with lung ultrasonography. Lung ultrasonography was performed by placing the transducer on the third intercostal space along the midclavicular line on both sides of the chest for visualizing pleural sliding and lung expansion, which was synchronized with manual ventilation (Fig. 2). The tube tip was advanced gently into the right or left mainstem bronchus, until unilateral lung sound was lost, and pleural sliding and lung expansion was not visualized on one side of the lung on scanning. After confirming the endobronchial position of the tube tip, the tube was withdrawn slowly until bilateral lung sounds were auscultated and bilateral pleural sliding and lung expansion were visible, and then further withdrawn, so that the tube tip was positioned 2 cm above the carina The tube was secured to the lower lip and inflation of tube cuff was determined with the same methods as those used in the conventional group.

After positioning the tube, the neck was extended by 30°–45° and the head was placed on a pliable head collar with a small rolled pad under the neck for palatoplasty. A Dingman mouth gag was used for surgical field exposure. However, during cheiloplasty, a Dingman mouth gag was not used and the neck was positioned with 0°–10° extension.

After positioning the patient, the appropriate tube depth was rechecked by auscultation in the conventional group and using ultrasonography of the subglottic area and both the lungs in the ultrasound group. Mechanical ventilation was provided using 50% air in oxygen with 2–2.5% sevoflurane and gas flow rates of 1.5–2 L min^−1^ at the rate of 20–25 breaths min^−1^, with a tidal volume of 8–10 ml kg^−1^ to maintain end-tidal carbon dioxide at 30–45 mmHg. Intraoperative monitoring included heart rate, non-invasive blood pressure and axillary body temperature measurement, and electrocardiography, pulse oximetry, and capnography. Elective surgery was performed under adequate analgesia with a bolus dose of fentanyl 1 µg kg^−1^. Neuromuscular blockade was antagonized with pyridostigmine and glycopyrrolate at the end of surgery. The patient was extubated when fully awake with adequate spontaneous respiration and shifted to the postanesthetic care unit.

### Data management

Collected data included patients’ demographics and concurrent deformities. In the operation theater, the initially selected tracheal tube size, insertion depth, need for tube exchange to determine final tube size, and oxygen desaturation (pulse oximeter reading < 90%) were recorded. Ventilation indices including tidal volume, respiratory rates, peak airway pressure and end-tidal carbon dioxide, incidence of tube displacement including accidental extubation or endobronchial intubation, airway complications including bronchospasm (that is, oxygen desaturation combined with wheezing on auscultation and a prolonged expiratory slope on capnography), laryngospasm (that is, oxygen desaturation due to partial or complete airway obstruction relieved by positive pressure ventilation or muscle relaxant) and post-extubation stridor (that is, a new-onset inspiratory high-pitched sound after extubation), and total operation time were recorded. In the postanesthetic care unit, symptoms such as hoarseness, barking cough, and oxygen desaturation were recorded by an independent anesthesiologist who was blind to the method of tube selection.

### Study endpoint

The study’s primary outcomes included the incidence of airway complications and adverse events in the intraoperative and immediate postoperative periods. Complications and adverse events were classified as major if therapeutic intervention was needed, and minor, if only observation was needed. Major complications and adverse events included laryngospasm, bronchospasm, post-extubation stridor, and oxygen desaturation (< 90%) since re-intubation, tube displacement and disconnection. Minor complications and adverse events included hoarseness, barking cough, need for tube exchange, and any tube displacement that was corrected immediately without desaturation. The secondary outcomes included the initially selected tube size, insertion depth, ventilatory indices and total operation time.

Post hoc power analysis verified whether disparate sample size between the groups could achieve sufficient power for the primary endpoint, and revealed that sample sizes of 28 and 23 were enrolled in the conventional and ultrasound groups, respectively, to achieve 87% power for detecting differences between the group proportions of 0.278 under 4.3% of occurrence rate of complication in the ultrasound group and 32.1% in the conventional group. The one-sided Z test with pooled variance was used for statistical analysis with PASS 12 (NCSS, LLC, Kaysville, UT, USA). The significance level was set at 0.05 and 0.0507 was achieved by this design.

### Statistical analyses

Statistical analyses were conducted using the SPSS software version 25.0 (IBM Inc., Armonk, NY, USA). Continuous variables were expressed as the mean (standard deviation) or median (interquartile range, IQR [range]) and categorical variables were presented as the number (proportion), wherever appropriate. The independent Student t-test or Mann–Whitney U test was used to compare continuous variables, wherever appropriate. One-sided Fisher’s exact test was used to compare the incidence of airway complications and adverse events between the two groups because the alternative hypothesis was that the rate of complications would be lower in the ultrasound group than that in the conventional group. Other categorical variables were analyzed using Pearson’s chi-squared or Fisher’s exact test, wherever appropriate. Statistical significance was defined as *P* < 0.05.

## Results

Of the 51 patients included in the study, 28 were assigned to the conventional group and 23 to the ultrasound group. Patient baseline characteristics are summarized in Table 1. Demographic parameters, clinical diagnoses, and concurrent diseases or deformities were comparable for both the groups.

**Table 1:** Baseline characteristics of the study population.

Characteristic	Conventional group (n = 28)	Ultrasound group (n = 23)	*P* value
Sex, male	11 (39.3%)	14 (60.9%)	0.125
Age (months)	12.0 (4.0–14.3 [2.0–25.0])	12.0 (8.5–14.0 [3.0–27.0])	0.855
Height (cm)	75.1 (68.7–77.2 [45.0–97.2])	77.0 (71.0–80.1 [62.6–94.0])	0.368
Weight (kg)	9.4 (7.6–10.2 [5.5–12.5])	10.1 (8.5–10.8 [6.6–14.4])	0.191
**Clinical diagnosis**			0.147
Complete lip	4 (14.3%)	2 (8.7%)	
Incomplete lip	3 (10.7%)	2 (8.7%)	
Complete palate	0 (0%)	2 (8.7%)	
Incomplete palate	17 (60.7%)	11 (47.8%)	
Complete lip and palate	3 (10.7%)	2 (8.7%)	
Submucous cleft palate	1 (3.6%)	4 (17.4%)	
**Concomitant diseases and deformities**			0.054
Cardiac anomaly	8 (28.6%)	3 (13.0%)	
Pierre–Robin syndrome	1 (3.6%)	0 (0%)	
Cardiac anomaly and Goldenhar syndrome	1 (3.6%)	0 (0%)	

Values are number (proportion) or median (interquartile range [range]).

Intubation failure did not occur in either group. Table 2 summarizes the intraoperative outcome variables. No differences were observed in tracheal tube size, ventilatory indices and operation time of both the groups. Tube insertion depth (median (IQR) [range]) was significantly greater in the ultrasound group than in the conventional group (13.5 (12.5–14.0) [10.0–14.0] vs 13.0 (11.8–13.0) [11.0–14.0], *P* = 0.045).

**Table 2:** Intraoperative outcome variables.

Variable	Conventional group (n = 28)	Ultrasound group (n = 23)	*P* value
**Initial selected tracheal tube size (ID, mm)**			0.182
3.0	2 (7.1%)	0 (0%)	
3.5	10 (35.7%)	6 (26.1%)	
4.0	14 (50.0%)	15 (65.2%)	
4.5	2 (7.1%)	2 (8.7%)	
**Insertional depth (cm)**	13.0 (11.8–13.0 [11.0–14.0])	13.5 (12.5–14.0 [10.0–14.0])	0.045*
**Operation time (min)**	160 (138–180 [60–190])	160 (150–180 [30–190])	0.915
**Tidal volume (ml)**	93 (75–113 [50–160])	110 (80–120 [60–140])	0.377
**Respiratory rate (breaths min**^−1^**)**	22 (20–29 [18–35])	22 (19–23 [17–30])	0.165
**Peak airway pressure (cmH**_2_**O)**	20 (18–21 [15–29])	20 (18–21 [14–23])	0.605
**EtCO**_2_ **(mmHg)**	39 (36–41 [32–62])	38 (37–41 [32–57])	0.917
≤ 45	24 (85.7%)	20 (87.0%)	0.898
> 45	4 (14.3%)	3 (13.0%)	

Values are number (proportion) or median (interquartile range [range]).

*ID* inner diameter, *EtCO*_*2*_ end-tidal carbon dioxide.

*Statistically significant difference between the groups.

Table 3 summarizes the airway complications and adverse events. The incidence of complications and adverse events was higher in the conventional group than that in the ultrasound group (32.1% vs 4.3%, *P* = 0.013). Minor complications occurred in eight patients in the conventional group, including tube exchange, tube cuff herniation, endobronchial intubation caused by a tongue depressor, and cough and hoarseness in three, two, one, and two patients, respectively. Major complications, including post-extubation stridor followed by desaturation, occurred in only in one patient. In the ultrasound group, minor complications included tube exchange in one patient, while no major complications were observed.

**Table 3:** Airway complications and adverse events in the perioperative periods.

Complications and events	Conventional group (n = 28)	Ultrasound group (n = 23)	*P* value
**Total**	9 (32.1%)	1 (4.3%)	0.013*
**Minor complications**	8 (28.6%)	1 (4.3%)	
Tracheal tube exchange	3 (10.7%)	1 (4.3%)	
Tracheal tube cuff herniation	2 (7.1%)	0 (0%)	
Endobronchial intubation	1 (3.6%)	0 (0%)	
Cough and hoarseness	2 (7.1%)	0 (0%)	
**Major complication**	1 (3.6%)	0 (0%)	
Stridor	1 (3.6%)	0 (0%)	

Values are number (proportion).

*Statistically significant difference between the groups. *P* value was obtained using one-sided Fisher’s exact test.

Table 4 details the airway complications and adverse events according to the initially selected tracheal tube size in both the groups. Seven cases of events were related to the initially selected tube size in both the groups, except for three cases of tube cuff herniation and endobronchial intubation associated with insertion depth in the conventional group. Four cases of stridor, cough, and tube exchange due to resistance during tube insertion were related to relatively larger tube size, whereas two cases were related to relatively smaller tube size, including tube exchange due to an excessively audible air leak in the conventional group. The tube size was switched from an ID of 4.5–4.0 mm in one case in the ultrasound group, owing to resistance during intubation.

**Table 4:** Breakdown of airway complications and adverse events according to the initially selected tracheal tube size.

Tube size (ID, mm)	Conventional group			Ultrasound group		
	Total	Complications and events	Subdivision (no. of subjects)	Total	Complications and events	Subdivision (no. of subjects)
3.0	2	1	Tube exchange due to air leakage (1)	0	0	
3.5	10	3	Tube exchange due to air leakage (1) cuff herniation (1) stridor (1)	6	0	
4.0	14	5	Tube exchange due to resistance (1) cuff herniation (1) endobronchial intubation (1) cough and hoarseness (2)	15	0	
4.5	2	0		2	1	Tube exchange due to resistance (1)

Values represent the number of participants.

*ID* inner diameter, *No.* number.

## Discussion

The principal finding of this study was that tracheal tube size and insertion depth selection using airway ultrasonography in pediatric patients undergoing cleft repair surgery helped to decrease perioperative airway complications and adverse events.

We studied pediatric patients undergoing cleft repair surgery because they are vulnerable to airway morbidity, and the conventional age-based formula for tube selection is empirical and only used in patients aged 2 years and above^6–8^. Given that the airway diameter of infants under 12 months showed a poor correlation with demographic parameters including age, height, and weight, we expected that using airway ultrasonography for selecting tube size and insertion depth would reduce the airway and respiratory complications associated with tracheal tube-related issues in this population^17^.

Several studies have focused on tracheal tube size selection with ultrasound. The success rate of correct tube size selection based on airway diameter measurement ranges from 48 to 86%^14–16,18^. The wide variation in the success rate likely results from differences in ultrasound scanning levels and measuring points. The narrowest portion of the pediatric airway determined by imaging modalities remains controversial. The vocal cords and the area immediately below them are the narrowest parts in non-paralyzed children, as determined by magnetic resonance imaging and computed tomography^19,20^. The area just below the vocal cords is elliptical, whose transverse diameter is smaller than its anteroposterior diameter. While the transverse diameter increases linearly in the caudal direction, the anteroposterior diameter remains unchanged. The ratio of the transverse to anteroposterior diameters is 0.4, 0.5, and 0.8 at the vocal cord, subglottic, and cricoid levels, respectively. However, the vocal cords appear blurred on ultrasonography and can expand on muscle relaxation. Moreover, the area immediately below the vocal cords is ambiguous for ultrasound measurement. Contrarily, the cricoid cartilage is rigid, non-expandable, and more easily detectable than any other structure on ultrasonography. It appears as a round hypoechoic structure with hyperechoic edges, composed of perichondrium^15–17^. Given that the narrowest portion of the paediatric airway is not the cricoid cartilage but the vocal cords and subglottis above the cricoid cartilage, the air-mucosa interface within the cricoid cartilage is more suitable for selecting tracheal tube size using ultrasonography. Therefore, we chose the air-mucosa interface within the cricoid cartilage, and not the bilateral margins, as the scanning level and measuring point, providing more accurate information for tube size selection. In the ultrasound group, the tube selected using the subglottic transverse diameter was switched to a half-size smaller tube in one patient due to slight resistance immediately below the vocal cords during insertion. This may be attributed to the folding of the deflated cuff and the small predetermined margin of selection between the measured subglottic diameter and selected OD of tube, which was less than 0.5 mm.

The ideal patient age for surgery is approximately three months for cheiloplasty and 12 months for palatoplasty to ensure physiological and anatomical maturation. Estimation of actual airway size is difficult in patients with cleft anomalies, because their growth rate differs from that of normal children owing to nutritional deficiencies and recurrent infection. In our study, 80% (8/10) of airway complications and adverse events occurred in patients aged 12–14 months, owing to the selection of tubes with ID ranging from 3.5 to 4.0 mm. Therefore, patients in these age groups are more susceptible to airway complications and adverse events caused by inappropriate selection of tracheal tube size and insertion depth.

As the subglottic airway is elliptical rather than circular in shape, it is possible to detect an audible leak through the space above and below the tracheal tube, even if an adequately fitting tube is selected^21^. Two patients in the conventional group experienced cough and hoarseness. These results can be explained by a combined effect of the limitations of the air leakage test, and incorrect estimation of the tube size, inflation of cuff, and cuff position. Overestimation of tube size could exert significant pressure on the lateral wall of the subglottic airway. In contrast, underestimation of tube size would require greater inflation of the cuff for adequate sealing, increasing the pressure exerted by the cuff on the lateral wall of the elliptical airway and irritating the airway mucosa. Moreover, the intracuff pressure does not remain static but undergoes changes with neck positioning and cuff movement, even if it is within the predetermined acceptable range during initial cuff inflation^22^. As the distance from the vocal cords to carina is relatively short in infants, the cuff on the distal shaft of tube could inadvertently be displaced from the initial mid-tracheal level to the subglottic area after neck extension. Airway mucosal irritation resulting from cuff displacement, combined with over-inflation of the cuff, induced cough and hoarseness in the conventional group.

Positioning the tracheal tube tip correctly, including placing the cuff below the cricoid cartilage, is essential to prevent accidental extubation and reduce potential laryngeal damage from inappropriate cuff inflation. This is especially true for infants whose tracheal length is as small as 4.6–6.4 cm and the position of tracheal tube is influenced by the position of the head and neck^23,24^. Herniation of the cuff out of the vocal cords was observed in two patients in the conventional group during surgery. If the tube tip was assumed to be placed 2 cm above the carina by auscultation, the cuff of tube would move upward to the mid-trachea or higher in the subglottic region, especially if the neck is extended. Auscultation alone could not confirm cuff position. In contrast, tube insertion depth was significantly greater in the ultrasound group than in the conventional group. Ultrasonographic determination of insertion depth was more reliable and ensured distal placement of the cuff below the cricoid cartilage by checking the tube shaft in the subglottic area after positioning the patient. Herniation of the cuff was not observed in any patient. Placing the tube tip at the mid-tracheal level was not ideal for cuffed tubes in children during palatoplasty, owing to their short tracheal length.

This study has several limitations. First, the experience gained during the study and long recruitment period may have affected the outcomes, as the cleft lip and palate has become a rare disease and only a limited age-group was available for study enrolment. Second, while the cuff pressure required for sealing the trachea was limited to the acceptable range, the volume of cuff inflation and cuff displacement during surgery were not evaluated due to the restricted surgical field. Third, this single-center cross-sectional observational study had a small sample size. Multi-center randomized clinical trials with larger sample sizes are needed to acquire more information regarding the benefits of ultrasonography.

Taken together, airway ultrasound application could reduce airway complications and adverse events associated with the selection of appropriate tracheal tube size and insertion depth during cleft repair surgery.

## Data Availability

The datasets generated during and/or analyzed during the current study are available from the corresponding author on reasonable request.
